# P-2304. Prevalence and clinical features of cefepime heteroresistance among bloodstream isolates of Pseudomonas aeruginosa in patients with hematologic malignancy

**DOI:** 10.1093/ofid/ofae631.2457

**Published:** 2025-01-29

**Authors:** Stephanie L Egge, William R Miller, James S Lewis, Morgan Hakki

**Affiliations:** Oregon Health & Science University, Portland, OR; Houston Methodist Research Institute, Houston, TX; Oregon Health and Science University, Portland, Oregon; Oregon Health and Science University, Portland, Oregon

## Abstract

**Background:**

Cefepime (FEP) is a primary choice for the empiric treatment of febrile neutropenia in hematologic malignancy (HM) patients who are at high risk for *Pseudomonas aeruginosa* (PA) infection. However, heteroresistance (hR) of Gram-negative pathogens has been associated with treatment failure, and the prevalence and impact of FEP hR across PA isolates in HM patients is unknown. We assessed FEP hR prevalence and associated microbiologic and clinical factors across PA bloodstream infections (BSI) in our institution’s HM patients.

Figure 1:

**Figure:**
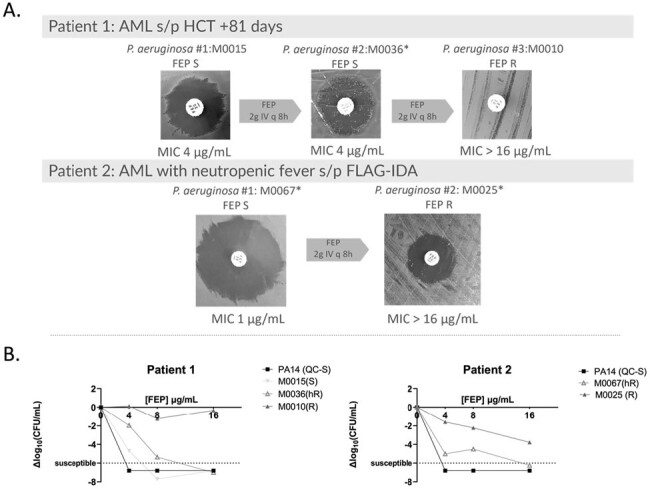

Clinical and microbiologic progression of two patients who developed breakthrough bacteremia on FEP therapy as seen by (A) standard clinical antimicrobial susceptibility assessment and (B) population analysis profile. * indicates hR per PAP-AUC.

**Methods:**

We retrospectively analyzed clinical data and isolates from 23 HM patients diagnosed with FEP susceptible PA BSIs at our institution from 2011-2023. Broth microdilution (BMD) minimum inhibitory concentration (MIC) and population analysis profile-area under the curve (PAP-AUC) were performed on index isolates and any subsequent BSI isolates while on FEP treatment. hR was defined as < 6log drop in growth at FEP PA MIC breakpoint (16 µg/mL) or PAP-AUC > 79, the 99% confidence interval predicted cut-off for PA14. Correlations between clinical data and susceptibility phenotype were assessed by Fischer-exact analyses and t-tests.

Table 1:

**Figure:**
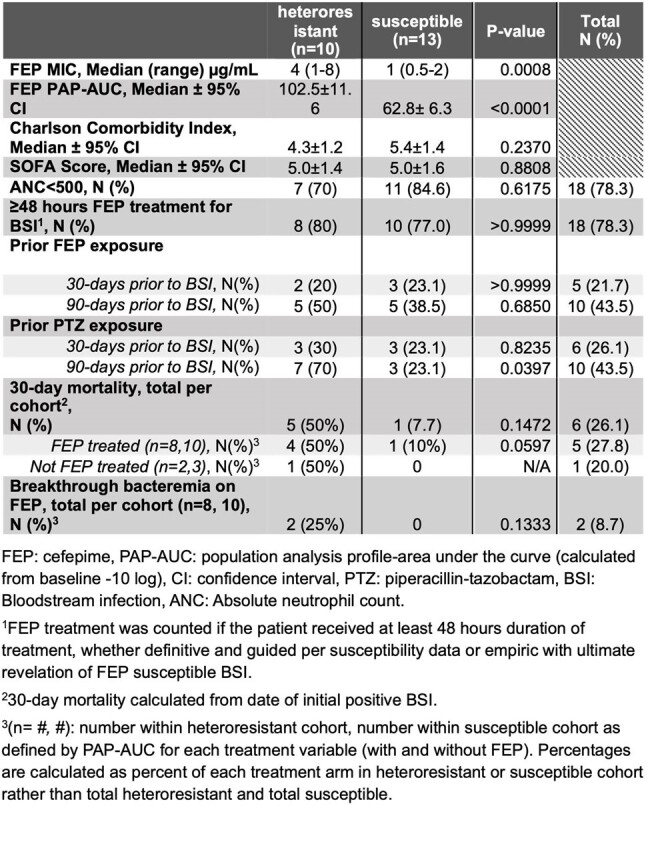

Clinical and microbiologic characteristics of FEP hR and non-hR P. aeruginosa bloodstream isolates in HM patients

**Results:**

Twenty-three index isolates had confirmed susceptible MICs ≤ 8 µg/mL (Table 1) per BMD. Ten of 23 (43%) isolates met criteria for hR. FEP MICs for hR isolates were significantly higher than susceptible isolates (P=0.0008, Table 1). There was no difference between prior FEP exposures among hR and non-hR isolates, but prior piperacillin-tazobactam (PTZ) exposure correlated with FEP hR (Table 1, P=0.04). Despite equal morbidity and severity presentation amongst cohorts, there was a trend towards increased 30-day mortality amongst patients with FEP hR PA BSI (P=0.06) treated with FEP. Two patients with FEP hR BSIs developed breakthrough bacteremia on FEP with FEP-R isolates (Figure 1); both died in the setting of recurrent PA BSIs.

**Conclusion:**

FEP hR is prevalent in HM patients with PA BSIs, particularly isolates with MICs ≥ 4µg/mL. Our study showed numerically higher mortality in HM patients with BSIs due to FEP hR isolates treated with FEP, and prior PTZ exposure may increase risk of BSI with an FEP hR isolate. Further studies are needed to determine prevalence and clinical impact of FEP hR in this vulnerable population.

**Disclosures:**

William R. Miller, M.D., Merck: Grant/Research Support|UptoDate: Royalties James S. Lewis, PharmD, FIDSA, Entasis: Advisor/Consultant|La Jolla pharmaceuticals: Advisor/Consultant|Merck: Advisor/Consultant

